# Renewable energy and ecological footprint nexus: Evidence from dynamic panel threshold technique

**DOI:** 10.1016/j.heliyon.2024.e33442

**Published:** 2024-06-22

**Authors:** Mohammad Naim Azimi, Mohammad Mafizur Rahman

**Affiliations:** School of Business, University of Southern Queensland, QLD, 4350, Australia

**Keywords:** Renewable energy, Ecological footprint, Human development, Institutional quality, Population density

## Abstract

The escalating phenomenon of environmental degradation is an urgent global concern, imperiling ecosystems and hindering the prospects for sustainable development on a planetary scale. Therefore, this study aims to explore the intricate interplay between renewable energy (RE) and ecological footprint (EF), considering the conditional impact of fiscal capacity (FIC), human development (HDI), institutional quality (IQI), and population density (PDN). Drawing on panel data encompassing 74 developing countries from 2000 to 2022, the study employs a dynamic panel threshold regression method, both with and without an instrumental variable approach. The findings unveil a non-linear nexus between RE and EF, revealing significant threshold values for FIC (1.870), HDI (0.736), and IQI (0.311), above which RE showcases its efficacy in mitigating EF. Conversely, when these predictors dip below the thresholds of FIC (1.391), HDI (0.655), and IQI (0.2545), the impact of RE on FE becomes insignificant. Moreover, the study introduces PDN as an additional threshold variable in the analysis, pinpointing that the effectiveness of RE in reducing EF hinges on PDN being below a threshold value of 263.144; however, above a threshold value of 276.98, the influence of PDN on the RE-FE nexus diminishes. The findings underscore the complexity of policy landscapes in developing countries. They suggest that while promoting renewable energy is pivotal for environmental sustainability, it is equally imperative to bolster existing environmentally friendly fiscal capacity, advance human capital, enhance institutional quality, and craft effective population distribution policies.

## Highlights

• This study examines a panel of 74 developing countries from 2000 to 2022.
• It reveals asymmetric effects of RE on EF.
• FIC above a certain level significantly influences the impact of RE on EF.
• Low levels of HDI and IQI do not effectively mitigate EF through RE.
• Below a certain threshold, PDN enhances the relationship between RE and EF.

## Graphical abstract

**Figure:**
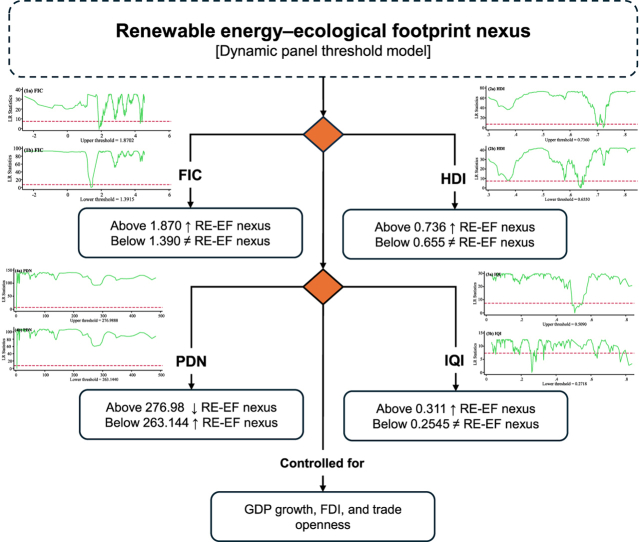

## 1 Introduction

Environmental degradation has emerged as pressing global challenge, with escalating levels of CO_2_ emissions (CO_2_e) and ecological footprint (EF) posing significant threats to environmental sustainability [1]. This trend not only jeopardizes the planet's ecological balance but also presents formidable obstacles to economic and human development. In particular, developing nations such as China, India, Indonesia, Bangladesh, Pakistan, Nigeria, and others despite being pivotal to achieving the Sustainable Development Goals (SDGs) by 2030, are grappling with severe environmental issues [2]. These nations, while significant contributors to pollution and CO_2_e [3,4], have yet to fully harness and invest in cleaner, renewable energy sources to drive sustainable environmental practices. The resource-intensive nature of development in these nations, aimed at rapid economic growth and poverty reduction, has inadvertently led to increased CO_2_e and EF. Despite the intention to prioritize green energy resources, the reality often falls short, resulting in significant environmental strain. Notably, the burgeoning populations in many developing countries exacerbate these challenges, compounding environmental threats to both citizens and their habitats. In urban areas and industrial hubs, characterized by capital- or labor-intensive manufacturing activities, there is a notable surge in conventional energy consumption, greenhouse gas emissions (GHGs), waste production, extensive water use, and altered land patterns [5]. These activities contribute substantially to EF, thereby deteriorating environmental quality and posing significant risks to sustainability.

The United Nations [6] has emphasized the pivotal role of renewable energy in steering nations towards sustainable environmental practices. To achieve this, it is proposed that renewable energy should constitute more than 60 % of the total energy demand by firms and individuals by the end of 2030 [7]. This critical shift aims to bolster contemporary industrial resilience and foster eco-friendly economic growth, particularly in developing economies. Given this imperative, there is a pressing need to delve into the intricate and yet unexplored dynamics of renewable energy's impact on EF. Understanding this nuanced relationship under specific conditions can shed light on critical areas of concern. Such insights can play a crucial role in guiding the recalibration of existing environmental policies in developing economies towards more sustainable and environmentally conscious practices.

While prior empirical studies have significantly contributed to our understanding of the impact of renewable energy on environmental indicators, EF in particular (refer [[8], [9], [10]]), investigations into the specific conditions governing the symmetric and asymmetric effects of renewable energy on EF remains substantially scarce or even non-existent, as far as our knowledge extends. This empirical gap in the existing literature serves as a key motivation for our current piece of research, as we aim to provide novel insights that can reshape the perspectives of environmental and economic policymakers in developing nations. Indeed, the diverse environmental contexts and the wide range of social and economic threats linked to environmental challenges, including inadequate enforcement of environmental protection regulations, high economic volatility, insufficient economic infrastructure, rapid population growth, unsafe environmental practices, poverty, and ongoing process of industrialization [11], emphasize the importance of directing our focus towards developing nations. These factors have propelled us to select developing countries as the focal point of our study. Additionally, beyond the intrinsic importance of understanding the dynamics within the developing nations, the decisions made regarding environmental sustainability at the national level can have far-reaching implications. The environmental conditions affecting neighboring nations and potentially reverberating across the international community. Compared to prior literature that has merely examined the contemporaneous and symmetric relationships between renewable energy and environmental indicators, our study takes a novel approach by exploring the threshold effects of renewable energy on EF. This investigation employs fiscal capacity, human development, institutional quality, and population density as critical threshold variables, recognizing their relevance in the context of obstacles to sustainable environmental quality in developing economies. Through this novel approach, we aim to pinpoint the intricate and complex interplay between renewable energy and EF within the unique settings of large panels of developing countries. To address these objectives, the study formulates three pertinent research questions on contemporary environmental issues: Firstly, does renewable energy effectively mitigate EF across a spectrum of developing nations? Secondly, are there discernible threshold effects of key predictors that elucidate the improvement or hindrance of EF? Thirdly, what do fiscal capacity, human development, institutional settings, and population density influence the relationship between renewable energy and EF in developing nations? Answering these questions offers invaluable empirical insights into environmental quality, providing crucial guidance for the formulation of effective environmental policies in developing nations.

This study significantly contributes to the existing body of knowledge in several key aspects. Firstly, it employs a novel panel dynamic threshold regression analysis with and without an instrumental variable approach to examine the effects of renewable energy on EF. This method allows us to uncover the true effects of renewable energy on EF while addressing any potential endogeneity concerns that may arise in the regressions. Second, the study utilizes a comprehensive panel dataset encompassing 74 developing countries over a 23-year period. This extensive data set ensures robust estimations and allows for reliable conclusions to be drawn. Thirdly, the study introduces a novel approach by constructing a composite index for institutional quality using a wide range of institutional measures. This index provides a nuanced understanding of the threshold effect of institutional quality on the relationship between renewable energy and EF. By establishing minimum quality benchmark for institutional performance, this technique highlights specific areas where attention is crucial for macro-environmental policies. Fourthly, through the estimation of multiple threshold regressions considering fiscal capacity, human development, and population density, the study sheds light on how each threshold measure influences the impact of renewable energy on EF. This analysis offers insights into the optimal levels of eco-friendly fiscal capacity, required human capacity, and maximum population density conducive to improving environmental quality through renewable energy consumption. From a policy perspective, these findings provide valuable guidance on setting thresholds for enhancing environmental sustainability in developing countries.

The remainder of this study is structured as follows: Section 2 reviews the relevant available literature. Section 3 outlines the model design, data period, sources of compilation, definition, and selection of variables. Section 4 presents the empirical results. Section 5 presents a brief discussion. Section 6 concludes the study and discusses specific policy implications, limitations, and future research directions.

## 2 Literature review

Environmental degradation, prominently manifested by EF and CO_2_e, is intricately linked to a plethora of socio-economic indicators. A substantial body of research has sought to unravel the complex web of connections between environmental degradation and a diverse array of potential drivers. In this section, we delve into the extensive literature, aiming to distill key insights relevant to the context of this study.

### 2.1 EF-renewable energy nexus

The interplay between renewable energy and EF is complex, influenced by the dynamic behaviors of human activities within the environmental sphere [[12], [13], [14], [15]]. Usman and Hammar [16] investigated the impact of renewable energy on EF alongside financial development, growth, and population factors in Asia Pacific Economic Cooperation (APEC) countries, analyzing data from 1990 to 2017. Their findings observed that the utilization of renewable energy notably enhances environmental quality by decreasing EF levels across their recipient panel. Conversely, Raghutla et al. [8] delved into the effects of renewable energy on EF across N-11 from 1990 to 2018. Their study, employing both quantile regression and long-run elasticities, revealed that renewable energy adoption, alongside per capita income, significantly contributes to the increase in EF. Additionally, Sharma et al. [17] examined the short- and long-run effects of renewable energy, population density, and life expectancy on EF across eight South and Southeast Asian countries from 1990 to 2015, employing the cross-sectional augmented autoregressive distributed lag (CS-ARDL) technique. The authors demonstrated that the heightened utilization of renewable energy significantly diminished EF in the region. Usman et al. [18] explored the interplay between renewable energy innovation and EF in G-7 nations using the method of moments quantile regression approach from 1985 to 2016. Their study confirmed the validity of the Environmental Kuznets Curve (EKC) hypothesis and revealed that renewable energy innovation has mitigating effects on EF. Likewise, Xue et al. [19] explored the effects of renewable energy on EF in four South Asian nations, namely Bangladesh, India, Pakistan, and Sri Lanka, using the CS-ARDL model. Their findings indicated that renewable energy reduces EF, whereas nonrenewable energy increases it. The study supported the validity of the EKC and pollution haven hypotheses. Destek and Sinha [20] applied the EKC framework to examine the effects of renewable energy on EF across 24 Organization for Economic Cooperation and Development countries from 1980 to 2014. Employing the mean group approach, their findings revealed a U-shaped relationship between economic growth and EF, concluding that an increase in renewable energy leads to a reduction in EF. Furthermore, Raza et al. [21] investigated the impact of technology innovation and renewable energy on EF in G20 countries from 1990 to 2021. Using the CS-ARDL model, their findings revealed that renewable energy and technological innovation have negative impacts on EF. Although the contributions of prior studies are invaluable, the mixed results presented necessitate to capture the true effects of renewable energy on EF across diverse economies. To address this gap, we propose the following hypothesis:

H1 Renewable energy, in conjunction with major macroeconomic indicators, exhibits a threshold effect on EF.

### 2.2 EF-human development nexus

The existing literature offers a diverse range of perspectives on the relationship between EF and human development, lacking a definitive consensus on the magnitude of the effects of human development on EF. Chen et al. [22] investigated the impact of human capital on EF on a global scale, analyzing data from a large panel of developing countries from 1990 to 2016. Their findings revealed an interesting pattern where human capital primarily contributed to an increase in EF, followed by a subsequent reduction. Likewise, Ahmed and Wang [23] explored the effects of human development on EF in India from 1971 to 2014. Using the cointegration test of Bayer and Hanck, their results suggested a negative association between human development and EF, establishing a causal link from human development to EF. Moreover, Opoku et al. [24] examined the effects of human development on EF across OECD countries from 1996 to 2016, employing the Driscoll-Kraay and panel quantile regression methods. Their results revealed that an increase in human development contributes to enhanced environmental sustainability, manifested through reductions in EF, CO_2_e and GHGs. Conversely, Saleem et al. [25] investigated the influence of human development on EF, CO_2_e, N_2_O, and CH_4_ across BRICS member countries from 1991 to 2014. Employing the dynamic seemingly unrelated regression approach, their findings indicated that while human development plays a significant role in reducing EF in developed economies, its impact becomes insignificant in developing countries, resulting to a controversial effects of on EF. Furthermore, Danish et al. [26] explored the interplay of human development on the effects of economic growth on EF in Pakistan using the ARDL method from 1971 to 2014. Their results demonstrated that an increase in human activities, leading to economic growth, resulted in an increase in EF n Pakistan. While existing research highlights the diverse impact of human development on EF, its role as a threshold and moderating factor remains unexplored. To offer a nuanced understanding, we develop the following hypothesis:

H2 Human development acts as a threshold moderator in the EF-renewable energy relationship.

#### 2.2.1 EF-institutional quality nexus

Institutional quality is a macroeconomic factor that primarily remained extensively unexplored in the context of its impact on EF [27,28]. The review reveals that Iorember and Yusoff [29] examined the role of governance quality and renewable energy transition in the income–environmental quality nexus in 34 African nations from 1996 to 2018, using a series of advanced panel techniques. Their findings confirmed the escalating impact of income on CO_2_e, while the interactive term of renewable energy transition and per capita income had significant negative effects on CO_2_e. Crucially, their results support the significance of governance quality on reducing emission levels. Additionally, Goel et al. [30] investigated the impact of institutional quality on environmental degradation across 100 nations from 2004 to 2007. Their study focused on corruption and the shadow economy as key factors. The results indicated that both corrupt nations and those with significant shadow sectors exhibited similar adverse effects on environmental quality, leading to higher levels of pollution. Additionally, Azimi and Rahman [31] examined the impact of institutional quality on EF in G20 countries from 2000 to 2022 using a limited information maximum likelihood panel analysis. Their results demonstrated a substantial role of institutional quality in reducing EF across G20 nations. Moreover, they found that the transparency index had a particularly strong influence on curbing EF. Fatima et al. [32] employed dynamic panel quantile regression to examine the effect of globalization, institutional quality, and growth on environmental degradation in OCED countries from 1991 to 2018. Their results revealed that globalization and institutional quality have negative affect on environmental degradation, whereas growth has a positive impact. Furthermore, Hunjra et al. [33] examined the moderating role of institutional quality on the relationship between financial development and environmental quality in South Asia from 1984 to 2018. Their findings indicate that while financial development tends to increase environmental degradation, institutional quality moderates this negative impact, thus supporting environmental sustainability. Abaidoo and Agyapong [34] explored the effect of institutional quality on EF in Sub-Saharan Africa using the limited information maximum likelihood technique. Their study revealed that institutional quality mitigates the adverse effects of urbanization on EF. Likewise, Uzar [35] investigated the impact of institutional quality on EF in E-7 countries from 1992 to 2015 using the augmented mean group technique. Their findings revealed that institutional quality decreases EF across the panel. Furthermore, the effect of institutional quality on EF varies across countries. Although prior research has made valuably contributions to the existing body of knowledge, the specific threshold effects of institutional quality on EF in developing nations have not been extensively explored. To address this gap, we propose the following hypothesis:

H3 Institutional quality demonstrates threshold effects, influencing the relationship between EF and renewable energy.

#### 2.2.2 EF-growth nexus

The link between economic growth and EF has been the focal point in prior literature. For instance, Shahbaz et al. [36] examined the impact of economic growth on EF across various countries such as China, USA, India, Japan, Brazil, Indonesia, Mexico, Korea, Turkey, and UK from 1992 to 2017, using the common correlated effects method. They observed that growth, alongside non-renewable energy, exerts a negative effect on environmental quality, leading to an increase in EF. In the same vein, Lee et al. [37] delved into the interplay between EF and growth in OECD countries from 1995 to 2017, employing a panel quantile regression approach. Their study revealed that growth initially led to an increase in EF, yet in later stages of growth, it contributed to a decrease in EF, thereby confirming the EKC hypothesis. Majeed et al. [38] investigated the asymmetric effects of energy consumption and growth on environmental quality in Pakistan from 1971 to 2014, finding that positive shocks to oil and gas consumption increased EF, while negative shocks decreased it. Among others, a leading study by Iorember et al. [39], analyzed data spanning from 1990 to 2019 across BRICS nations using the CS-ARDL model and Tapio decoupling index method to investigate whether they are successfully reducing CO_2_e while maintaining economic growth. Their findings provided evidence indicating that these nations commenced to decouple growth from emissions, with Brazil leading the progress, followed by Russia and South Africa, while India and China exhibited slower progress. Additionally, Çakmak and Acar [40] studied the relationship between growth, renewable energy, and EF in oil-producing nations from 1999 to 2017. Their findings supported the Pollution Haven Hypothesis, showing a significant impact of growth on EF, while renewable energy consumption did not exhibit a significant effect. Moreover, Vu et al. [41] investigated the dynamic relationship among growth, globalization, financial inclusion, and EF in the Middle-East and North Africa (MENA) from 1990 to 2017. Their study revealed significant direct impacts of financial inclusion, growth, and globalization on EF through GDP, while ecological innovation was found to decrease EF in the MENA region. Acar et al. [42] examined the dynamic relationship between growth, EF, and financial development in Azerbaijan from 1996 to 201, using the ARDL bound test with structural breaks. Their findings indicated that growth tends to increase EF, whereas financial development exhibits a mitigating effect, reducing EF. Similarly, Udemba [43] explored the nexus between growth and EF in Nigeria through the ARDL and Granger causality techniques. The authors observed a parallel rise: as economic growth increases, so does EF, showing that economic growth propels further EF in the country. While prior studies have mainly assumed the contemporaneous links between growth and EF, its conditional impacts remained unexplored. To capture this gap, we propose the following hypothesis:

H4 The effects of growth on EF vary in magnitude and size, contingent upon the threshold effects of contributing factors.

#### 2.2.3 EF-population nexus

The dynamic effects of population on environmental degradation has been fairly studies in prior literature [44,45]. It is widely recognized that population growth and density have significant and often adverse impacts on environmental quality. For example, Weber and Sciubba [44] delved into the spatial effects of population growth on environmental degradation across 1062 regions relevant to 22 European countries between 1990 and 2006. Their results not only supported the presence of spatial autocorrelation in population distribution but also confirmed the significant impact of population growth on environmental degradation. Additionally, Dimnwobi et al. [46] examined the dynamics of population on EF in Congo, Ethiopia, Nigeria, South Africa and Tanzania from 1990 to 2019 using the CS-ARDL. Their study revealed that, along with economic and social indicators, population density was found to have a detrimental effect on environmental quality, leading to an increase in EF. Furthermore, Kongbuamai et al. [47] explored the impact of economic growth, energy consumption, and population density in Thailand from 1974 to 2016. Employing the ARDL bounding test and vector autoregressive technique, they observed that alongside growth and energy consumption, population density also contributed to the deterioration of environmental quality, leading to an increase in EF. Conversely, Hussain et al. [48] examined the dynamics of population density on environmental quality in Pakistan from 1981 to 2016, utilizing the EKC framework and the ARDL model. Their results suggested a negative and statistically significant impact of population density on EF, indicating that population density does not contribute to environmental degradation. Furthermore, Anser et al. [49] investigated the influence of population density on EF across a large panel of 130 countries from 1995 to 2018. The authors employed the GMM technique and observed that population density has a detrimental impact on EF. Additionally, they noticed a bidirectional causality between EF and population density across their recipient panel. While existing research has predominantly focused on the unconditional impact of population density on environmental degradation, the results presented are mixed. Therefore, to assess the threshold moderating effects of population density on the relationship between EF and renewable energy, we propose the following hypothesis:

H5 Population density has a threshold effect on the EF-renewable energy nexus.

## 3 Methodology

### 3.1 Selection of sample and data

Given the paramount global concern of escalating environmental degradation, particularly poignant in developing economies, this study focuses on a sample of developing economies as identified by the World Bank report [50]. The study period spans from 2000 to 2022, dictated by data availability for the variables of interest to ensure a robust panel for a precise evaluation. Consequently, we curated the panel, excluding countries lacking sufficient data, resulting in a final selection of 74 out of 152 countries. The list of countries used in the study is presented beneath Table 1. Additionally, Table 1 details the sources from which the data has been compiled.

**Table 1:** Variables, symbols, and sources.

Variables' name	Symbol	Unit of measurement	Sources of compilation
Ecological footprints	EF	Per capita; consumption	GFN [80]
Renewable energy consumption	RE	% of the total energy consumption	WDI [81]
Fiscal capacity	FIC	% of total taxes and grants to the GDP ratio	UNU-WIDR [82]
Human development index	HDI	Numbers ranging from 0 (low) to 1 (high)	UNDP [83]
Institutional quality index	IQI	Numbers ranging from 0 (low) to 1 (high)	WGI [84]
Population density	PDN	Number of individuals per square kilometer of land area	WDI [81]
Gross domestic product growth rate	GDPG	Annual growth rate, %	WDI [81]
Foreign direct investment	FDI	% of GDP	WDI [81]
Trade openness	TOP	% of GDP	WDI [81]

Notes: GFN: Global Footprint Network, WDI: World Development Indicators, WGI: Worldwide Governance Indicators, UNDP: United Nations Development Program, UNU-WIDR: UN University-World Institute for Development Economic Research.

Countries: Afghanistan, Albania, Algeria, Angola, Argentina, Armenia, Azerbaijan, Bangladesh, Belarus, Benin, Bhutan, Bolivia, Botswana, Bosnia & Herzegovina, Brazil, Burkina Faso, Burundi, Cameroon, Chad, China, Colombia, Congo, DR Congo, Costa Rica, Cuba, Dominican Republic, Ethiopia, Gambia, Ghana, Guatemala, Guinea, Guinea-Bissau, Haiti, India, Indonesia, Jamaica, Jordan, Kenya, Lebanon, Lesotho, Mali, Malawi, Madagascar, Malaysia, Mexico, Mongolia, Mozambique, Myanmar, Nepal, Nicaragua, Niger, Nigeria, Pakistan, Panama, Peru, Philippines, Poland, Romania, Sierra Leone, Slovakia, Sri Lanka, Suriname, Syria, Thailand, Togo, Tunisia, Turkey, Turkmenistan, Uganda, Uzbekistan, Venezuela, Vietnam, Yemen, Zambia.

### 3.2 Selection of variables

#### 3.2.1 Dependent variable

Prior literature has commonly employed *CO*_2_e as a measure of environmental degradation in various nations [[51], [52], [53], [54]]. However, in this study, we use consumption-based ecological footprint (EF) as a proxy for environmental degradation primarily driven by human interactions. The EF metric gauges the bio-productive use of resources such as fisheries, livestock, cropland, etc., supporting human and business needs. Thus, aligned with our study objectives, EF offers a more precise measure of the total resources utilized by humans and firms, potentially leading to environmental strain [[55], [56], [57], [58]].

#### 3.2.2 Independent variables

Our focus lies in examining the impact of renewable energy on EF under certain conditions, shedding light on key areas of concern within existing environmental policies. As such, we use renewable energy (RE) as the independent variable, measured as a percentage of total final energy consumption. While prior studies conducted by Raghutla et al. [8], Sahoo and Sethi [9], Nathaniel and Khan [10], Alola et al. [59], and Xue et al. [19] have also explored the relationship between RE and EF, they have primarily considered the contemporaneous link between these variables.

#### 3.2.3 Threshold variables

Due to its objectives, the study employs four key independent variables to examine their threshold effects on EF. Fiscal capacity (FIC), represented as a percentage of total taxes and grants to GDP ratio, serves as one of the threshold variables. FIC measures the resource-generated outcomes of population's economic interaction, influencing environmental quality [60]. As taxes on natural resources are considered inherent to a country and directly contribute to social development, we have excluded them from our analysis to avoid unnecessary effects on EF. Prior to our investigation, Ouedraogo et al. [61] were among the few to consider the effects of FIC. Additionally, the study incorporates the human development index (HDI) from the United Nations Development Program (UNDP) to capture its threshold effects on EF. The HDI, ranging from 0 (low) to 1 (high), encompasses all elements relevant to our investigation, measuring overall living standards, skills, knowledge development, and health of human capital. Similar proxies have been employed by Iskandar [62], Yolanda [63], Khan et al. [64], and Iqbal et al. [65] to gauge the impacts of human development on various socio-economic and environmental factors. Furthermore, the study employs a comprehensive aggregate index for institutional quality (IQI) to measure how its threshold impacts influence EF within the recipient panel. This index aids in highlighting specific areas where policymakers should focus to align existing environmental policies with sustainable environmental guidelines across developing nations. The methodology used for constructing IQI aligns with the approach suggested by Sarma [66], utilizing six governance measures from the Worldwide Governance Indicators (WGI). This technique, preferred for its empirical robustness and ease of estimation, has been widely adopted by prior literature [31,[67], [68], [69], [70]]. The resulting IQI is expressed as a number between 0 (low) and 1 (high), with detailed construction information available in Appendix A. Finally, the study includes population density (PDN), measured as the number of individuals per square kilometer of land area, as a threshold variable. Higher PDN typically correlates with increased EF. Emphasizing the importance of understanding the threshold effects of PDN on the relationship between RE and EF [71,72].

#### 3.2.4 Control variables

To ensure a precise evaluation, the study includes control variables to account for the effects of key macroeconomic factors on EF. The GDP annual growth rate (GDPG) is employed to capture the impact of economic variations on the subject. Similarly, prior studies conducted by Jahanger et al. [73], Shahbaz et al. [36], Ahmed et al. [74], and Baloch et al. [75] has consistently shown the significant influence of economic growth on contemporary levels of ecological footprint. Moreover, as the flow of financial capital and trade often leads to increased human interaction, resulting in higher EF, the study incorporates foreign direct investment (FDI) and trade openness (TOP) as percentages of annual GDP to trace their effects on EF. While the findings are mixed, studies conducted by Wang et al. [76], Zubair et al. [77], Saqib et al. [78], and Mahadevan and Sun [79] have provided evidence of the significant impact of FDI and TOP on EF across the studied panel.

### 3.3 Model design

Building on prior literature, there have been limited attempts to explore the effects of RE on EF in the presence of various endogenous predictors [37,55,[85], [86], [87]]. In this study, we contribute to the existing body of knowledge by incorporating FIC, IQI, HDI, and PDN predictors in our empirical analysis. It is crucial to emphasize how the exogeneity of environmental quality influences concurrent policy directions in developing economies. The empirical framework of our analytical model is based on the foundational works of Levine and Zervos [88] and King and Levine [89], who developed their growth models in the 1990s. This approach has since gained prominence in empirical analysis across various topics. Given its rationality, accuracy, and policy orientation nature, we adopt a similar methodology for our analysis as expressed in equation (1) below:

(1) $$EF_{i}=\beta^{\prime }RE_{i}+\gamma X_{i}+\varepsilon_{i}$$

where $EF_{i}$ represents the ecological footprint in country i, ${RE}_{i}$ denotes the country's level of renewable energy consumption, X represents the vector of other explanatory variables (FIC, IQI, HDI, PDN, GDPG, FDI, and TOP), and $\varepsilon_{i}$ is the error term. To scrutinize the array of hypotheses outlined in Section 2 (literature review), the study posits that equation (2) is predominantly adept at capturing contingency effects and providing a nuanced modeling of how fiscal capacity, institutional quality, human development, and population density influence the impact of renewable energy consumption in ecological footprint. Therefore, we adopt the threshold regression approach proposed by Hansen [90] to delve into the nonlinear dynamics of renewable energy consumption concerning ecological footprint. The model, structured on threshold regression, is expressed as follows:

(2) $$EF_{i}=\left(\beta_{1}RE_{i}+\gamma_{1}X_{i}\right)I\left(K_{i}\leq \lambda \right)+\left(\beta_{2}RE_{i}+\gamma_{2}X_{i}\right)I\left(K_{i}\geq \lambda \right)+\varepsilon_{i}$$

where $K_{i}$ (*i.e.,* FIC, IQI, HDI, and PDN) represents the threshold variables used to partition our sample into regimes, and λ denotes the unknown threshold parameter. I(∙) represents the indicator function, taking a value of 1 if the argument in the indicator function holds and 0 otherwise. This approach allows the influence of renewable energy to vary depending on whether FIC, IQI, HDI, and PDN are above or below some unknown levels of λ. In this model, the impact of RE on EF is represented by $\beta_{1}$ for nations in the low-, and $\beta_{2}$ for nations in high-regimes. When the hypotheses $\beta_{1}=\beta_{2}$ and $\gamma_{1}=\gamma_{2}$ hold, equation (2) becomes linear [91] and reduces to (1). This approach has been widely utilized in prior literature due to its robustness and computational simplicity (refer [[92], [93], [94]]).

According to Bai [95] and Bai and Perron [96], the successive estimator is reliable, thus our initial estimation process involves testing the linearity assumption, denoted as $\beta_{1}=\beta_{2}$, against the threshold model (see equation (2)). As λ, the threshold parameter, is not identifiable under the null, posing a non-standard implication and the LM or Wald tests do not hold traditional $\chi^{2}$ limits [90], we calculate LM or Wald statistics for each possible values of λ using supremum approach [92]. Certainly, in this context, given the complexity of the tabulation and the non-standard distribution of supremum statistics relying on model-specific nuisance parameters, inferences are drawn using a model-based bootstrap approach. The validity and properties of this method were established by Hansen [90], offering a robust and effective means of conducting the necessary statistical inferences. However, despite the many advantages of panel data estimations in macroeconomic policy analysis, they are prone to certain empirical challenges, notably cross-sectional dependence (CD) and endogeneity issues. In addressing these issues, the study employs the instrumental variable (IV) threshold regression technique, as suggested by Caner and Hansen [97]. This approach rectifies problems related to CD and endogeneity, providing unbiased and consistent estimates, particularly for any reverse causal effects, under a valid IV assumption. To implement this, we adjust equation (2) as follows:

(3) $$EF_{i}=\left(\beta_{1}RE_{i}+\gamma_{1}X_{i}\right)I\left(K_{i}\leq \lambda \right)+\left(\beta_{2}RE_{i}+\gamma_{2}X_{i}\right)I\left(K_{i}>\lambda \right)+\varepsilon_{i}$$

(4) $$RE_{i}=\left(\vartheta_{1}Z_{i}+\omega_{1}X_{i}\right)I\left(K_{i}\leq \lambda \right)+\left(\vartheta_{2}Z_{i}+\omega_{2}X_{i}\right)I\left(K_{i}>\lambda \right)+\mu_{i}$$

where all vectors and variables have been defined before and $Z_{i}$ represents the vector of IVs. Importantly, equation (4) assumes that the threshold variables, such as FIC, IQI, HDI, and PDN, of course, are dealt with separately as exogenous variables. As suggested by Caner and Hansen [97], we follow a three-step approach to estimate equation (4). First, ${RE}_{i}$ will be regressed on $Z_{i}$ using the pooled ordinary least square (POLS) model to obtain the ${\hat{RE}}_{i}$ (say, the fitted values of RE). Next, ${\hat{RE}}_{i}$ will be substituted into equation (3) instead of ${RE}_{i}$ to estimate the threshold level λ. Third, using the estimated values of λ, we split the panel into perhaps two unequal sub-panels and compute the slope coefficients through the generalized method of moments (GMM) of Blundell and Bond [98] model, as in Caner and Hansen [97]. Finally, to examine the presence of a threshold impact, we use the Supremum Wald statistics (*S*-Wald-statistics) approach and the bootstrapping technique to derive the required asymptotic probability values for *S*-Wald-statistics [99,100]. These estimations are robust and performed using STATA-17 and R-programming software.

## 4 Results

### 4.1 Summary statistics

Table 2 provides essential descriptive statistics for the study variables. The mean value of EF is 1.971 per capita, notably higher than the global average of 1.51 per capita in 2022 [80]. Additionally, the mean value of RE is 42.671 % of the total final energy consumption in the recipient panel. Considerably, the mean value of FIC is 0.044 %, which is significantly low with a dispersion value of 0.090 from the mean value. HDI and IQI both have mean values of 0.609 and 0.288, showing that both are quite low in developing countries. However, as one can read through, the mean value of PDN stands at 119.531 and a maximum of 1301.039 individuals per square kilometer in land areas in developing countries. Among others, the PDN in developing countries is at its peak, being the highest density in Bangladesh (1301.039) in 2021 compared to the global average of 61 people per square kilometer of land area.

**Table 2:** Descriptive statistics, 2000 to 2022.

Variable	Observations	Mean	Standard deviation	Minimum	Maximum
EF	1702	1.971	1.212	0.442	7.766
RE	1702	42.671	31.917	0.050	98.340
FIC	1702	0.044	0.090	−0.007	0.891
HDI	1702	0.609	0.144	0.218	0.881
IQI	1702	0.288	0.219	0.003	0.967
TOP	1702	80.977	39.351	20.964	252.249
GDPG	1702	2.438	4.658	−29.922	33.030
FDI	1702	4.346	7.277	−40.086	106.594
PDN	1702	119.531	159.704	1.559	1301.039

Notes: EF: Ecological footprint, RE: Renewable energy, FIC: Fiscal capacity, HDI: Human development index, IQI: Institutional quality index, TOP: Trade openness, GDPG: Gross domestic product growth, FDI: Foreign direct investment, PDN: Population density.

**Fig. 1:**
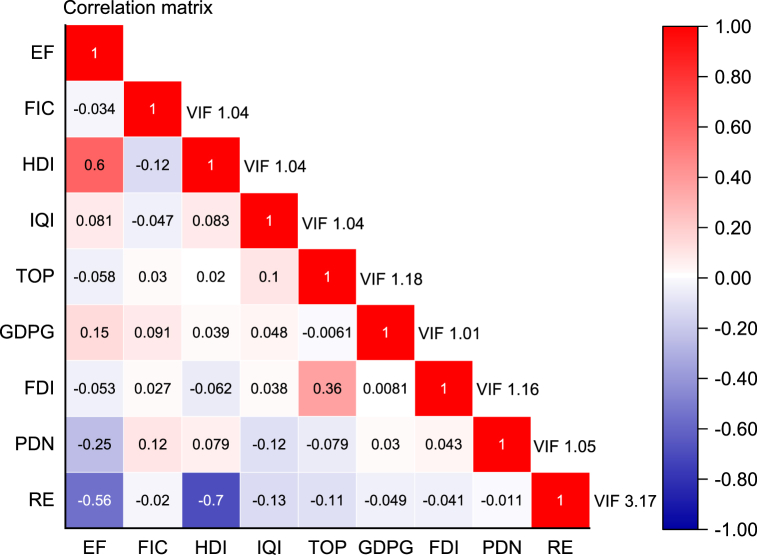
Correlation matrix.

Source: Authors' estimations.

**Fig. 2:**
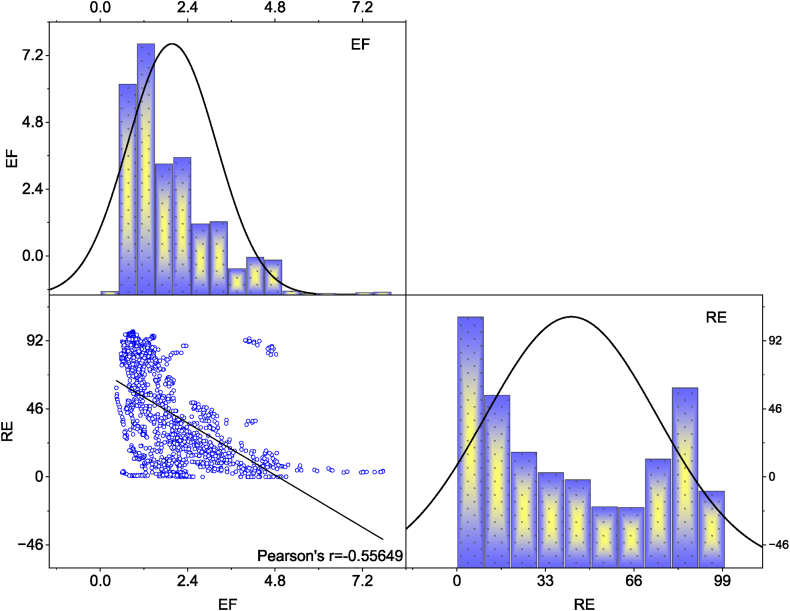
RE-EF correlation plot.

Source: Authors' estimation.

Additionally, Fig. 1 depicts the correlation between the variables, aiming to assess potential multicollinearity. Perfect or extreme multicollinearity can introduce bias in panel regression [101]. We apply two methods to test the existence of multicollinearity issues. First, following Elith et al. [102], a correlation of 0.85 or higher, regardless of the sign, indicates extreme multicollinearity among the variables. Our results in Fig. 1 reveal no signs of extreme multicollinearity. Second, individual variance inflation factors (VIFs) are observed to be low below 5.0. A VIF exceeding 5.0 indicates extreme multicollinearity [103]. Thus, our variables exhibit statistical soundness, enabling further analysis, as outlined in the preceding section. Finally, Fig. 2 illustrates the overall relationship between renewable energy and ecological footprint, showing a strong and negative association.

The study proceeds by estimating the cross-sectional dependence (CD) test proposed by Pesaran [104] and the second-generation panel unit root test introduced by Pesaran [105], known as the cross-sectionally augmented Im, Pesaran, and Shin (CIPS) method. The results presented in Table 3 reveal that, except for FIC, which lacks significance in rejecting the null hypothesis of no CD, the remaining variables exhibit significance in rejecting the null. This suggests a robust presence of cross-sectional dependence among the variables. The existence of CD among units commonly stems from homogenous economic structures, trade activities, and typical emission patterns [106,107]. Additionally, in the presence of CD, traditional panel unit root tests may fail to accurately capture the true stationarity of the variables. To address this limitation, the study employed the CIPS unit root test and reported the outcomes underneath the CD results in Table 3. The analysis reveals that, following the difference process, variables such as RE, PDN, and TOP demonstrate significance in rejecting the null hypothesis no-stationarity. Conversely, other variables including EF, IQI, HDI, GDPG, FDI, and FIC are significant at the level to reject the null of non-stationarity. This suggests that the variables follow mixed integration orders of I (0) and I (1).

**Table 3:** CD and CIPS results.

Models estimated	EF	RE	FIC	HDI	IQI	PDN	PGDP	FDI	TOP
CD-test	20.46***	38.20***	−0.78	218.89***	79.09***	171.81***	62.41***	15.06***	26.71***
p-value	0.000	0.000	0.435	0.000	0.000	0.000	0.000	0.000	0.000
CIPS unit root									
Level	−2.220***	−1.994	−2.424***	−2.101**	−2.679***	−1.507	−3.566***	−3.330***	−1.265
First-difference	−4.977***	−4.161***	−4.980***	−3.782***	−5.051***	−4.310***	−5.596***	−5.417***	−4.016***

Notes: *** indicates significance at a 1 % level. Critical values for CIPS at 1 %, 5 %, and 10 % levels are −2.17, −2.07, and −2.01, respectively.

### 4.2 Threshold regression analysis

Considering the mixed integrating orders of the variables presented in Table 3, dynamic panel threshold regression is often used to examine non-linear relationships between variables that change depending on certain trigger points [108]. This is useful even with variables exhibiting different integration orders, as it helps identify how they interact under varying conditions [97,109]. Subsequently, we apply equation (2) to scrutinize the threshold effects concerning our four threshold variables. Models 1–4 rigorously test the null hypothesis for the FIC, HDI, IQI, and PDN variables, respectively, with detailed outcomes delineated in Table 4. The associated *p-values* of the LR statistics are computed via a robust bootstrapping technique, employing 500 replications alongside 10 % trimming procedure. Upon the rejection of the null of no threshold effects, the significance of *p-values* across all the estimated models underscores the imperative to split the panel into two distinct samples of below and above the threshold values.

**Table 4:** Threshold estimates of FIC, IQI, HDI, and PDN.

	Model 1 FIC = Fiscal capacity		Model 2 HDI = Human development index	
	Statistics	Bootstrap p-value	Statistics	Bootstrap p-value
First sample split				
LM test for *Ho*	46.911***	0.000	23.467***	0.001
Threshold levels				
Lower threshold level	1.3915***	0.000	0.6550***	0.000
Upper threshold level	1.8702***	0.000	0.8360***	0.000
Second sample split				
LM test for *Ho*	9.201	0.325	10.067	0.310

	Model 3 IQI = Institutional quality index		Model 4 PDN = Population density	
	Statistics	Bootstrap p-value	Statistics	Bootstrap p-value
First sample split				
LM test for *Ho*	19.833***	0.002	21.008***	0.001
Threshold levels				
Lower threshold level	0.2545***	0.009	263.1440***	0.000
Upper threshold level	0.3110***	0.002	276.9888***	0.000
Second sample split				
LM test for *Ho*	7.561	0.427	9.447	0.355

Notes: ***, and ** indicate significance at 1 % and 5 % levels, respectively. LM: Lagrange multiplier. *Ho (null hypothesis)*: There is no threshold effect. LM: Lagrange multipliers. FIC: Fiscal capacity, HDI: Human development index, IQI: Institutional quality index, PDN: Population density.

**Fig. 3:**
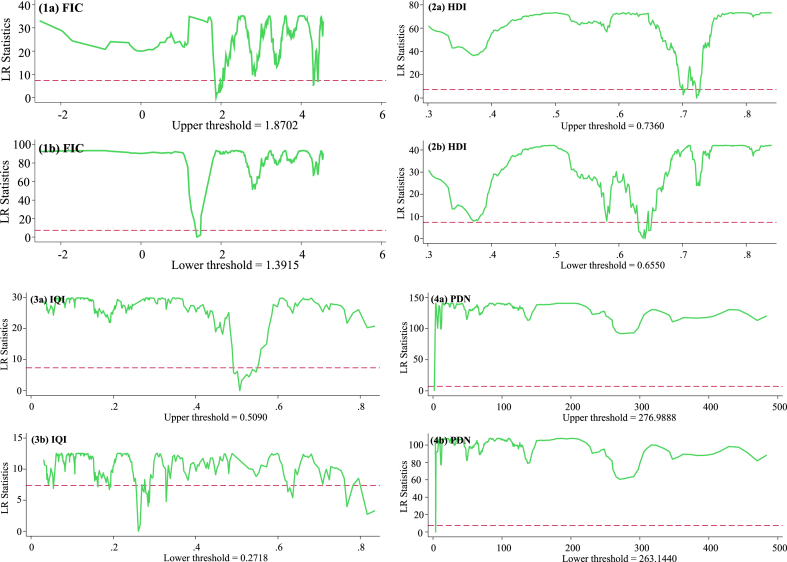
Estimated threshold levels.

Source: Authors' estimations.

While our findings yield statistical significance for the first regime or sample across models 1–4, the p-values were deemed statistically insignificant to warrant null hypothesis rejection in the second regime or sample. Consequently, we proceed by estimating a single-threshold model for all four key variables of interest, utilizing two sub-panels for values below and above the estimated threshold values. The estimated threshold values for FIC are 1.3915 and 1.8702 at the lower and upper levels, respectively. Likewise, the threshold level for HDI is 0.6350 at the low level and 0.7536 at the high level. Regarding IQI, the estimated threshold values at lower and upper levels are established to be 0.2545 and 0.3110, respectively. Finally, for PDN, the lower- and upper-level threshold values are 263.144 and 276.9888 people per square kilometer of land area, respectively. Moreover, we depict the threshold regime effects of the indicators in Fig. 3 for models 1–4.

After confirming the threshold effect values for the variables of interest in models 1–4, as illustrated in Fig. 3, the study proceeds to examine the impact of FIC, HDI, IQI, and PDN on the nonlinear relationship between RE and EF in the panel under review. Initially, we estimate the fixed effects model with robust standard errors proposed by Driscoll and Kraay (DK-FE) [110] without incorporating any threshold effects. This allows us to determine the average unconditional effects of the variables on EF for comparative analysis. Subsequently, we estimate the threshold regression model presented in equation (2), employing FIC, HDI, IQI, and PDN as threshold variables. The results are outlined in Table 5. In Table 5, one column is allocated to displaying the coefficients estimated below a threshold value, while another column reports the coefficients estimated above the threshold value for each model.

**Table 5:** Threshold regression results.

	DK-FE without threshold effect	Threshold regression model (1)		DK-FE without threshold effect	Threshold regression model (2)	
		FIC <1.3915	FIC >1.8702		HDI <0.6550	HDI >0.7360
Renewable energy	−0.0487*** (−4.16)	−0.015 (−1.29)	−0.196*** (−3.87)	−0.0017*** (−3.41)	−0.022 (−0.63)	−0.08906*** (−3.29)
Trade openness	−0.0215** (−2.29)	−0.009*** (−4.03)	−0.043** (−3.91)	−0.0328*** (−3.28)	−0.0013 (−0.94)	−0.0571*** (−4.46)
GDP growth rate	0.4599*** (3.67)	0.137*** (5.87)	0.108** (2.15)	0.224*** (4.42)	0.458*** (4.09)	−0.0088*** (−5.18)
Foreign direct investment	0.0129 (1.13)	0.10006* (1.94)	0.062** (2.48)	0.0188 (0.99)	−0.00007 (−1.11)	−0.0029*** (−3.29)
Fiscal capacity	−0.318*** (−3.98)			−0.269*** (−4.06)	−0.0054 (−0.68)	−0.0945*** (−5.67)
Human development index	−0.744*** (−10.33)	−0.227 (−1.16)	−0.911*** (−4.87)	−0.501*** (−4.18)		
Institutional quality index	−0.640*** (−4.00)	−0.611 (−0.35)	−0.704*** (−3.39)	−0.713** (−2.09)	0.000011 (1.06)	−0.0076** (−2.03)
Population density	0.024*** (5.48)	0.019*** (3.33)	0.039** (2.11)	0.01009** (3.15)	0.016* (1.82)	0.094*** (3.38)
Constant	0.871*** (4.40)	0.975*** (7.61)	0.942*** (9.88)	1.027*** (10.02)	0.645*** (6.54)	0.633*** (6.92)
Adjusted r-squared	0.439	0.501	0.427	0.614	0.522	0.498
F-statistics	82.68***	29.93***	34.12***	31.29***	28.76***	40.13***
Observations	1,702	1,127	575	1,702	943	759
Number of groups	74	49	25	74	41	33

	DK-FE without threshold effect	Threshold regression model (3)		DK-FE without threshold effect	Threshold regression model (4)	
		IQI <0.2545	IQI >0.3110		PDN <263.144	PDN >276.9888
Renewable energy	−0.0416*** (−3.49)	−0.00025 (−0.29)	−0.0708*** (−3.11)	−0.0333*** (−3.36)	−0.00100 (−1.17)	−0.0919*** (−4.63)
Trade openness	−0.0244* (−1.86)	−0.067 (−1.01)	−0.0915*** (−4.20)	−0.0091*** (−3.29)	−0.0550 (−0.90)	−0.1077*** (−3.26)
GDP growth rate	0.1066*** (4.26)	0.2544*** (−4.21)	0.0163*** (−3.39)	0.0604*** (3.67)	0.1966** (2.44)	0.4025*** (−4.10)
Foreign direct investment	0.0118 (0.59)	0.011 (0.28)	−0.1098*** (−3.72)	0.1207 (1.14)	0.0167 (1.32)	0.0207 (0.73)
Fiscal capacity	−0.259*** (−4.02)	−0.0018 (−0.74)	−0.361*** (−3.46)	−0.349*** (−3.95)	−0.2009 (−0.58)	−0.2318*** (−3.49)
Human development index	−0.601*** (−6.29)	−0.111 (−1.15)	−0.915*** (−4.02)	−0.663*** (−4.12)	−0.1077 (−1.19)	−0.947*** (−4.93)
Institutional quality index	−0.729*** (−3.47)			−0.548*** (−3.50)	−0.255*** (−3.14)	−0.02807 (−1.10)
Population density	0.0311*** (3.91)	0.029*** (4.84)	0.0630*** (4.33)	0.0387*** (3.66)		
Constant	0.922*** (9.15)	0.844*** (11.03)	0.939*** (11.18)	0.810*** (7.48)	0.909*** (8.64)	0.897*** (10.15)
Adjusted r-squared	0.546	0.499	0.468	0.619	0.582	0.511
F-statistics	74.25***	80.45***	76.43***	58.77***	41.99***	40.13***
Observations	1,702	1,150	552	1,702	1,035	667
Number of groups	74	50	24	74	45	29

Notes: ***, **, and * indicate significance at 1 %, 5 %, and 10 % levels, respectively. t-statistics are presented in parentheses. DK-FE indicates Driscoll and Kraay fixed effects model. FIC: Fiscal capacity, HDI: Human development index, IQI: Institutional quality index, PDN: Population density.

In model (1), the estimated coefficient of RE is statistically insignificant in influencing EF when FIC falls below the estimated threshold value. However, RE becomes highly significant in reducing EF when FIC surpasses the threshold level. These findings suggest that there is a nonlinear relationship between EF and RE in developing countries. Specifically, as RE consumption increases due to FIC forces, EF decreases in an asymmetric manner by 0.196 per capita. These insights are pivotal for shaping environmental policy decisions. The discerned nonlinear relationship between renewable energy (RE) consumption and ecological footprint (EF) in developing countries, especially when the FIC exceeds the threshold value, underscores the urgent necessity for significant investment in renewable energy. This investment is not only vital for enhancing environmental quality but also for advancing sustainable development objectives. Achieving this goal necessitates more than just domestic resources—it requires strong international cooperation efforts and cross-regional collaboration. These results align with the findings of Li et al. [111], Ullah et al. [112], Shabir et al. [113] Lee et al. [114], and Arif et al. [115], who have similarly observed asymmetric effects of RE on EF. Furthermore, the derived coefficients for TOP, GDPG, FDI, HDI, IQI, and PDN are in line with theoretical assumptions. The coefficients of TOP are statistically negative across all estimated models (1–4), regardless of whether above or below the FIC, HDI, IQI, and PDN threshold levels. However, below the estimated threshold levels, the effects of TOP are smaller than those above the threshold level. These findings are consistant with prior studies by Okelele and Lokina [116], Cutcu et al. [117], and Rehman et al. [56]. Specifically, Adebayo et al. [118] employed wavelet coherence and consistently identified a phase-out relationship between TOP and EF, noting that an increase in TOP leads to a decrease in EF. In contrast, the impact of GDPG on EF exhibits a positive trend. Below the FIC threshold level, GDPG exerts a more substantial influence, gradually diminishing beyond this threshold level. Notably, our findings show that within the context of developing countries, the effect of GDPG on EF undergoes a reduction from 0.4599 (average unconditional impact) to 0.108 per capita, attributable to FIC dynamics. Consisting findings regarding the relationship between GDPG and EF have been documented in studies by Wang et al. [119], Danish et al. [26], Lee et al. [37], and Shahbaz et al. [36] across diverse nations. Additionally, consistent with our outcomes, Yang et al. [120] and Khan et al. [121] in a study encompassing a larger and more diverse sample, noted that economic growth exerts a detrimental impact on contemporary ecological footprint. This effect is primarily attributed to rapid industrialization and increased human interaction.

Moreover, the results indicate that the effects of FDI on EF remain statistically insignificant across all estimated models, except for model (3), wherein the presence of an elevated IQI above the threshold level renders FDI significant in reducing EF by 0.1098 per capita. It is observed that the net flow of FDI continues to hold significance in shaping environmental quality, emphasizing the critical role of FDI moderators in altering its effects on the environmental sphere [122]. These insights hold crucial implications for environmental policy formulation. Policymakers need to recognize the importance of enhancing institutional quality to effectively leverage FDI for environmental sustainability. Strategies aimed at improving governance, transparency, and regulatory frameworks in developing nations can help maximize the positive impact of FDI on environmental quality. Our findings also emphasize the imperative of integrating environmental considerations into FDI policies and fostering synergies between economic development and environmental sustainability agendas. While Khan et al. [123] and Hunjra et al. [33] employed CO_2_e as a proxy for environmental degradation, their results align with ours regarding the effects of institutional quality on the relationship between FDI and EF. However, in contrast to our findings, studies by Salahuddin et al. [124] and Shahbaz et al. [125] presented statistical evidence indicating that FDI has a positive effect in promoting environmental quality. Regarding HDI, its average unconditional effects are significant at the 1 % level, leading to a reduction in EF by 0.744 per capita. However, when using FIC as a threshold variable, the coefficient of HDI becomes insignificant below the threshold level but gains significance above the threshold level, with an effect size of −0.911 per capita. These findings suggest that EF strongly responds to the changes in HDI across models 1, 3, and 4 when using FIC, IQI, and PDN as threshold variables. This underscores the crucial role of human capital development in mitigating the contemporary levels of EF in developing countries. Thus, policymakers should consider integrating thresholds related to economic and institutional factors to effectively leverage human development for environmentally sustainable development. Our results align with prior studies conducted by Stylianou et al. [126], Pata et al. [127], Ahmed and Wang [23], Chen et al. [22], and Sultana et al. [128]. Notably, Ünal and Aktuğ [129] and Pata and Hizarci [130] have demonstrated that incorporating human development into environmental policies leads to a significant reduction in ecological footprint, addressing unsustainable human interaction within society.

Additionally, in models 1–3, the unconditional positive effects of PDN on EF average at 0.024 per capita. However, when FIC, HDI, and IQI are utilized as threshold variables, the effects of PDN on EF decrease to 0.019, 0.016, and 0.029, respectively, at the lower threshold. Conversely, at the upper threshold level, the impact of PDN on EF increases to 0.039, 0.094, and 0.063 per capita, respectively. These findings suggest that EF will significantly decrease if PDN drops below 263 people per square kilometer, while environmental quality will deteriorate if PDN increases above 276 people per square kilometer of land area in developing countries. The results suggest that policymakers must prioritize measures to manage population growth sustainably and mitigate associated environmental impacts. This could involve implementing policies to encourage family planning, promoting sustainable urbanization, and investing in efficient infrastructure and resource management systems to alleviate strain on natural resources. Conversely, if PDN drops below the lower threshold level, there's a potential for a significant reduction in EF, indicating an opportunity to improve environmental quality. In such cases, policymakers should focus on leveraging this demographic shift to implement conservation measures, invest in green technologies, and promote sustainable land use practices to foster environmental sustainability and enhance overall quality of life for their citizens.

These results align with recent studies conducted by Ohlan [131], Egbetokun [132], Apergis and Ozturk [133], and Sapkota and Bastola [134] highlighting the significance of population density in driving environmental degradation. Furthermore, except for RE, model (2) yields result that are largely identical to those estimated in model (1). The findings regarding the asymmetric relationship between RE and EF hold true when HDI is used as a threshold variable in model (2). Specifically, the results demonstrate that with a percentage change in RE, EF asymmetrically decreases by 0.089 per capita above the threshold level, while the effects of RE on EF are insignificant below the estimated threshold level. Consistent with models 1 and 2, the estimated coefficient of RE remains significantly negative at the 1 % level to reduce EF in the recipient panel above the threshold level when IQI and PDN are used as threshold variables. However, the coefficients of RE are insignificant below the estimated threshold values in models 3 and 4. Notably, when IQI is used as a threshold variable, the effects of RE are significant above the threshold value, reducing EF by 0.0708 per capita, while it reduces EF by 0.0919 per capita above the threshold level when PDN is used as a threshold variable. Essentially, natural resources and the environment may suffer because of high population density in a country. These stresses have the potential to cause deforestation, overcrowding, and the breakdown of fragile ecosystem that supports environmental quality. As large amounts of non-renewable resources, such as oil, coal, and lumber, are consumed by expanding populations, EF increases. These findings support the nonlinear relationship between EF and RE through IQI and PDN threshold effects. While prior studies conducted by Naimoglu [135], Mohammad et al. [136], Sinha and Shahbaz [137], Sugiawan and Managi [138], Destek and Sinha [20], Danish and Ulucak [139], Alola et al. [140], Qiao et al. [141], Adamas and Nsiah [142], Diallo [143], and Sharma et al. [17] have primarily on direct and unconditional effects, they have also supported similar effects of RE on environmental degradation. Furthermore, other variables retain similar signs of the coefficients with highly identical effects on EF in models 3 and 4.

### 4.3 IV-threshold regression

Using Caner and Hansen's [97] approach as shown in equation (4), we conducted *IV*-threshold regression analysis as a robustness test, reported in Table 6. Given the strong endogeneity of environmental degradation indicators, particularly CO_2_e and EF, and the existence of reverse causality nexus between RE and EF, caution is exercised. The presence of endogeneity could compromise the consistency and robustness of coefficients estimated via pooled ordinary least squares methods. To address this, we employ Hausman's [144] method for the full panel of 74 countries, which indicates significant endogeneity (J-statistics: 23.14; p-value: 0.000) and confirms reverse causality from EF to RE across all threshold variables. Consequently, we utilize the first lag of RE as an instrumental variable. Moreover, following the procedure outlined in the previous section, we compute threshold values for FIC, HDI, IQI, and PDN, and re-estimate models 1–4 using the two-step System-GMM model proposed by Blundell and Bond [98]. The results, presented under models 1a–4a in Table 6, provide additional insights into the robustness of our findings. The estimated threshold values for FIC, HDI, IQI, and PDN, as determined by Supremum Wald-statistics and validated by bootstrapping technique, are statistically significant at a 1 % level. This reaffirms the validity of threshold effects even in the presence of endogeneity and CD in the panel, prompting the partitioning of panels into sub-panels below and above the threshold levels, as previously done. The results obtained from IV-regression analysis under models 1a–4a in Table 6 closely resemble those from threshold regression estimates reported in Table 5 under models 1–4, regarding the magnitude and significance of variables' effects on EF, the dependent variable.

**Table 6:** IV-threshold regression results.

	S-Wald-stat. *p-value*	Threshold regression model (1a) 51.381*** 0.000		Threshold regression model (2a) 25.442*** 0.003	
		FIC <1.389	FIC >1.891	HDI <0.667	HDI >0.742
Renewable energy		−0.019 (−0.88)	−0.194*** (−4.00)	−0.024 (−1.12)	−0.0912*** (−4.18)
Trade openness		−0.011*** (−3.94)	−0.045** (−3.51)	−0.0010 (−1.10)	−0.0566*** (−4.37)
GDP growth rate		0.135*** (3.48)	0.110*** (3.37)	0.467*** (3.87)	−0.0091*** (−4.40)
Foreign direct investment		0.0967 (0.66)	0.061*** (3.29)	−0.00015 (−0.66)	−0.0023*** (−4.31)
Fiscal capacity				−0.0062 (−1.25)	−0.0937*** (−6.01)
Human development index		−0.231 (−1.21)	−0.905*** (−3.46)		
Institutional quality index		−0.614 (−1.1)	−0.712*** (−3.27)	0.0043 (0.59)	−0.0081*** (−4.00)
Population density		0.017*** (4.03)	0.036*** (4.41)	0.019*** (3.24)	0.091*** (5.17)
Constant		0.805*** (10.22)	0.999*** (8.37)	0.814*** (8.45)	0.810*** (9.11)
Arellano and Bond (1)		−5.067***	−4.055***	−3.982***	−4.032***
Arellano and Bond (2)		−1.019	−0.976	−1.244	−1.097
Sargan-Hansen *J*-stat.		18.49***	21.37***	25.01***	20.14***
Observations		943	759	1,012	690
Number of groups		41	33	44	30

	S-Wald-stat. *p-value*	Threshold regression model (3a) 64.018*** 0.000		Threshold regression model (4a) 59.711*** 0.000	
		IQI <0.271	IQI >0.324	PDN <259.27	PDN >275.619
Renewable energy		−0.0014 (−1.13)	−0.0710*** (−4.33)	−0.0015 (−0.55)	−0.0944*** (−3.08)
Trade openness		−0.090 (−1.19)	−0.0918*** (−3.19)	−0.0538 (−0.82)	−0.1029*** (−3.59)
GDP growth rate		0.385*** (−3.74)	0.016*** (−4.21)	0.1962*** (3.29)	0.4103*** (−3.67)
Foreign direct investment		0.209 (1.21)	−0.110*** (−3.85)	0.0158 (1.17)	0.0214 (1.06)
Fiscal capacity		−0.036 (−1.04)	−0.35806*** (−3.29)	−0.2012 (−0.74)	−0.2336*** (−3.88)
Human development index		−0.317 (−0.98)	−0.921*** (−3.97)	−0.1056 (−0.99)	−0.955*** (−4.12)
Institutional quality index				−0.248*** (−4.10)	−0.034 (−0.87)
Population density		0.044*** (3.95)	0.071*** (4.59)		
Constant		0.927*** (8.14)	0.844*** (9.22)	0.918*** (8.27)	0.715*** (9.66)
Arellano and Bond (1)		−3.981***	−4.022***	−4.111***	−4.009***
Arellano and Bond (2)		−0.805	−1.149	−0.505	−1.319
Sargan-Hansen *J*-stat.		16.00***	20.20***	19.86***	16.32***
Observations		874	828	920	782
Number of groups		38	36	40	34

Notes: ***, and ** indicate significance at 1 % and 5 % levels, respectively.

Specifically, the threshold effects of FIC (1.389 below; 1.891 above), HDI (0.667 below; 0.742 above), IQI (0.271 below; 0.324 above), and PDN (259.27 below; 275.618 above) are statistically significant at the 1 % level, supported by corresponding p-values of the Supremum Wald-statistics being <0.01. Furthermore, the threshold effects of RE exhibit a similar pattern as shown in Table 5. For instance, when utilizing FIC as a threshold variable, RE does not influence EF below the threshold level, but becomes significant above the threshold value, reducing EF by 0.194 per capita. Similarly, under HDI and IQI thresholds, RE is statistically significant above the threshold level, reducing EF by 0.0912 and 0.0710 per capita, respectively. Finally, when using PDN as a threshold variable, RE is statistically significant in reducing EF by 0.0944 per capita above the threshold value. Other variables such as GDPG, TOP, and FDI demonstrate similar behavior, as depicted in Table 5. To ensure the robustness of models 1a–4a, important diagnostic tests are conducted and reported underneath each model in Table 6. For instance, the null hypothesis of second-order autocorrelation is rejected by Arellano and Bond (AR2) statistics at a 1 % level, indicating that the estimated models are free from autocorrelation. Additionally, the Sargan-Hansen J-statistics are highly significant for all estimated models, confirming the validity of over-identification restrictions on the instrumental variable used in estimating each model. Consequently, the results are robust, allowing us to proceed with highlighting significant and novel insights in the subsequent section.

## 5 Discussion

Environmental quality does not change automatically; it is influenced by multiple social, economic, political, and demographic factors. We approach this phenomenon from a novel perspective. Thus, in this empirical investigation, we explore the asymmetric and complex relationship between ecological footprint and renewable energy consumption, considering various social, economic, political, institutions, and demographical indicators such as fiscal capacity, human development, institutional quality, and population density in a panel of 74 developing countries. However, the generalizability of the overall results still holds; the number of selected countries was primarily based on the availability of the required data. The results presented in this study strongly advocate the need for the promotion and utilization of renewable energy in developing countries to mitigate environmental degradation by reducing ecological footprint. However, the threshold values highlight the minimum level of renewable energy consumption necessary for effectively improving environmental quality. In essence, the results imply that developing countries, which endorse rapid economic growth to boost their contemporary economic output, can leverage sources such as hydropower, bioenergy, solar energy, wind power, etc., to mitigate environmental hazards. They also underscore that the development and utilization of renewable energy contributes to environmental sustainability in line with SDG-7 under certain conditions.

Once the threshold effects have been verified (Table 4), our findings suggest that the nonlinear effects of renewable energy on ecological footprint persist when using key variables such as fiscal capacity, human development index, institutional quality index, or population density as thresholds (Table 5). Specifically, under the influence of fiscal capacity, human development, institutional quality, and population density, renewable energy emerges as a critical determinant of ecological footprint reduction in developing countries beyond certain levels. These findings underscore specific policy implications crucial for environmental policymaking. For instance, our research reveals that enhancing environmentally friendly fiscal capacity, aimed at improving government efficiency and economic performance, fosters the development and consumption of renewable energies, leading to a decline in ecological footprint. Similar recommendations are echoed in recent studies by Georgiou [60] and Ahmad and Satrovic [145]. Additionally, human development plays a pivotal role in shaping contemporary environmental quality in developing countries. Our findings highlight the decisive impact of the human development index above a certain threshold level. We demonstrate that even a modest improvement in the human development index can significantly influence the efficacy of renewable energy in reducing ecological footprints (Table 5, Table 6; 2 and 2a). These results align with studies by Ahmed et al. [72], Lan and Munro [146], Abdouli and Omri [147], Çakar et al. [148], and Jain and Nagpal [149].

While concerted efforts to increase the share of renewable energy consumption may partially mitigate ecological footprints and enhance environmental quality in developing countries [[150], [151], [152]], it's imperative to acknowledge the role of institutional quality. Recognizing this, we innovatively utilize a comprehensive institutional quality index as a threshold variable and find that improvements in institutional quality above a certain benchmark render renewable energy consumption more effective in reducing ecological footprints (Table 5, Table 6; models 3 and 3a). This is consistent with findings by Christoforidis and Katrakilidis [153], Uzar [35], Hussain and Dogan [154], and Xu et al. [155]. Moreover, population density poses significant environmental challenges in developing countries. Enriching the literature on this topic, studies by Hussain et al. [48], Sharma et al. [17], and Nadee et al. [156] emphasize the impact of population density on ecological footprints. Utilizing population density as a threshold variable, we demonstrate that reducing population density below a certain threshold level, such as 263 people per square kilometer of land area, enhances the efficacy of renewable energy consumption in reducing ecological footprints (Table 5, Table 6; models 4 and 4a). Furthermore, our analysis reveals a positive effect of GDP growth and a negative impact of trade openness on ecological footprints, indicating a trade-off between growth policies and environmental degradation in developing countries. While the interaction between the institutional quality index and foreign direct investment is significant, our results do not support the effects of foreign direct investment on ecological footprints in other vector models, with and without threshold effects (Table 5, Table 6).

## 6 Conclusion

Employing a panel of 74 developing countries over a period of 23 years from 2000 to 2022, this study explores the effects of renewable energy consumption under certain threshold effects of fiscal capacity, human development, institutional quality, and population density on ecological footprints. Datasets sourced from reliable sources were compiled, and a novel institutional quality index was constructed for precise assessment. The analysis utilized Hansen's threshold model [90] and the proposed instrumental variable threshold technique of Caner and Hansen [97] to address any endogeneity issues throughout the estimations. Preliminary results reveal significant threshold effects of fiscal capacity, human development, institutional quality, and population density, underscoring the need to split the panel into sub-panels based on these variables for a comprehensive understanding of the relationship between renewable energy and ecological footprints.

After estimating the threshold values for each indicator, the results indicate an asymmetric relationship between renewable energy and ecological footprint under the threshold effects of fiscal capacity, human development, institutional quality, and population density. Renewable energy demonstrates effectiveness in reducing ecological footprints above certain threshold levels, while its effects are statistically insignificant below the estimated threshold level. This suggests that in developing countries, the significance of renewable energy in reducing ecological footprints depends on advancing fiscal capacity, improving human development capacity to interact with the environment, enhancing concurrent institutional quality, and reducing existing population density. Importantly, similar findings are consistently obtained from the instrumental variable threshold model, underscoring the robustness and accuracy of the study's outcomes.

### 6.1 Policy implications

The findings of this study underscore several specific areas where policy considerations can improve environmental quality:

a. Fiscal re-adjustment: Improved fiscal capacity can facilitate the use of renewable energy to promote environmental quality in developing countries. Policymakers should integrate environmental outcomes into macroeconomic strategies, emphasizing simultaneous environmental and economic growth. Additionally, incentivizing environmental quality as a public good and imposing prices on contamination is another alternative policy measure.
b. Human development: Human interaction is crucial in an environment. Renewable energy can only be used to reduce environmental degradation when population capacity reaches a certain level of development. Poor environmental human behavior, whether in terms of combustion or negligence in protecting environmental goods, is a critical macro-policy area. Policymakers should, in fact, pay closer attention to promoting contemporary human development capacity in developing economies through knowledge, skills, awareness, and incentive measures.
c. Institutional quality: Developing countries need to uplift the quality of their institutions. Our findings suggest that institutional quality plays a critical role in influencing the effects of renewable energy on the ecological footprint. Therefore, mitigating corruption, enforcing laws, achieving effective public organization performance, and advancing regulatory quality are major areas where attention is sought to protect environmental quality in developing countries.
d. Population distribution: Population density is found to be a major player in increasing the ecological footprint in developing economies. Existing policies concerning population distribution seem to be ineffective and have caused catastrophic density. Policymakers need to ensure an environmentally friendly distribution through onshore and offshore business and employment shifts without compromising the existing population growth rate.
e. International cooperation: Given the global nature of environmental challenges, fostering international cooperation and collaboration is essential. Developing nations can benefit from sharing knowledge, expertise, and resources with developed nations to accelerate their transition towards sustainable energy systems. Collaborative efforts in technology transfer, capacity building, and financial assistance can help developing nations overcome barriers to renewable energy adoption and mitigate environmental degradation effectively.

### 6.2 Limitation and future directions

Due to fundamental empirical challenges, the study encounters two key limitations. Firstly, due to quality issues and extreme multicollinearity, the analysis necessitated the exclusion of the non-renewable energy consumption variable. Secondly, the unavailability of data precluded the examination of the effects of renewable energy segmentation on ecological footprint. Future studies could mitigate these limitations by addressing data availability constraints and exploring segment-specific measures for renewable energy in developing countries. Embracing a similar framework and methodology, futures research endeavors could provide insights into these aspects upon the availability of requisite data, potentially enriching our understanding of the relationship between renewable energy and environmental sustainability.

## Data availability statement

The datasets will be available upon reasonable request from the corresponding author.

## Funding

This work did not receive any funds from any organization.

## CRediT authorship contribution statement

**Mohammad Naim Azimi:** Writing – original draft, Validation, Software, Methodology, Formal analysis, Data curation, Conceptualization. **Mohammad Mafizur Rahman:** Writing – review & editing, Validation, Supervision, Project administration, Conceptualization.

## Declaration of competing interest

The authors declare that they have no competing financial interests to declare.

## References

[1] Ahmad M., Jiang P., Majeed A., Umar M., Khan Z., Muhammad S. (2020). The dynamic impact of natural resources, technological innovations and economic growth on ecological footprint: an advanced panel data estimation. Resour. Pol..

[2] Castells-Quintana D., Dienesch E., Krause M. (2021). Air pollution in an urban world: a global view on density, cities and emissions. Ecol. Econ..

[3] Lin J. (2014). China's international trade and air pollution in the United States. Proc. Natl. Acad. Sci. U.S.A..

[4] Wang F. (2020). China's trade-off between economic benefits and sulfur dioxide emissions in changing global trade. Earth's Future.

[5] Shen Y., Yue S. (2023). Does ecological footprint affect biocapacity? Evidence from the experiences of G20 countries. Nat. Resour. Model..

[6] United Nations (2023). https://press.un.org/en/2023/ecosoc7136.doc.htm.

[7] Zhao F., Zhang Y., Alharthi M., Zafar M.W. (2022). Environmental sustainability in developing countries: understanding the criticality of financial inclusion and globalization. Sustain. Dev..

[8] Raghutla C., Padmagirisan P., Sakthivel P., Chittedi K.R., Mishra S. (2022). The effect of renewable energy consumption on ecological footprint in N-11 countries: evidence from Panel Quantile Regression Approach. Renew. Energy.

[9] Sahoo M., Sethi N. (2021). The intermittent effects of renewable energy on ecological footprint: evidence from developing countries. Environ. Sci. Pollut. Res..

[10] Nathaniel S., Khan S.A.R. (2020). The nexus between urbanization, renewable energy, trade, and ecological footprint in ASEAN countries. J. Clean. Prod..

[11] Dhandapani K., Venkataraman H. (2023). The Palgrave Handbook of Global Sustainability.

[12] Shah W.U.H., Hao G., Yan H., Yasmeen R., Lu Y. (2023). Energy efficiency evaluation, changing trends and determinants of energy productivity growth across South Asian countries: SBM-DEA and Malmquist approach. Environ. Sci. Pollut. Res..

[13] Shah W.U.H., Hao G., Yan H., Zhu N., Yasmeen R., Dincă G. (2023). Role of renewable, non-renewable energy consumption and carbon emission in energy efficiency and productivity change: evidence from G20 economies. Geosci. Front..

[14] Yasmeen R., Zhang X., Tao R., Shah W.U.H. (2023). The impact of green technology, environmental tax and natural resources on energy efficiency and productivity: perspective of OECD Rule of Law. Energy Rep..

[15] Ul Hassan Shah W., Zhu N., Hao G., Yan H., Yasmeen R. (2024). Energy efficiency evaluation, technology gap ratio, and determinants of energy productivity change in developed and developing G20 economies: DEA super-SBM and MLI approaches. Gondwana Res..

[16] Usman M., Hammar N. (2021). Dynamic relationship between technological innovations, financial development, renewable energy, and ecological footprint: fresh insights based on the STIRPAT model for Asia Pacific Economic Cooperation countries. Environ. Sci. Pollut. Res..

[17] Sharma R., Sinha A., Kautish P. (2021). Does renewable energy consumption reduce ecological footprint? Evidence from eight developing countries of Asia. J. Clean. Prod..

[18] Usman O., Iorember P.T., Jelilov G., Isik A., Ike G.N., Sarkodie S.A. (2021). Towards mitigating ecological degradation in G-7 countries: accounting for economic effect dynamics, renewable energy consumption, and innovation. Heliyon.

[19] Xue L., Haseeb M., Mahmood H., Alkhateeb T.T.Y., Murshed M. (2021). Renewable energy use and ecological footprints mitigation: evidence from selected south asian economies. Sustain. Times.

[20] Destek M.A., Sinha A. (2020). Renewable, non-renewable energy consumption, economic growth, trade openness and ecological footprint: evidence from organisation for economic Co-operation and development countries. J. Clean. Prod..

[21] Raza A., Habib Y., Hashmi S.H. (2023). Impact of technological innovation and renewable energy on ecological footprint in G20 countries: the moderating role of institutional quality. Environ. Sci. Pollut. Res..

[22] Chen Y., Lee C.C., Chen M. (2022). Ecological footprint, human capital, and urbanization. Energy Environ..

[23] Ahmed Z., Wang Z. (2019). Investigating the impact of human capital on the ecological footprint in India: an empirical analysis. Environ. Sci. Pollut. Res..

[24] Opoku E.E.O., Dogah K.E., Aluko O.A. (2022). The contribution of human development towards environmental sustainability. Energy Econ..

[25] Saleem N., Shujah-ur-Rahman S.-R., Jun Z. (2019). The impact of human capital and biocapacity on environment: environmental quality measure through ecological footprint and greenhouse gases. J. Pollut. Eff. Control.

[26] Danish S. T. Hassan, Baloch M.A., Mahmood N., Zhang J.W. (2019). Linking economic growth and ecological footprint through human capital and biocapacity. Sustain. Cities Soc..

[27] Shah W.U.H., Hao G., Yan H., Yasmeen R., Padda I.U.H., Ullah A. (2022). The impact of trade, financial development and government integrity on energy efficiency: an analysis from G7-Countries. Energy.

[28] Azimi M.M., Rahman M.M. (2023). Impact of institutional quality on ecological footprint: new insights from G20 countries. J. Clean. Prod..

[29] Iorember P.T., Yusoff N.Y.M. (2023). Income–environmental nexus in Africa: the integrating role of renewable energy transition and governance quality. Afr. Dev. Rev..

[30] Goel R.K., Herrala R., Mazhar U. (2013). Institutional quality and environmental pollution: MENA countries versus the rest of the world. Econ. Syst..

[31] Azimi M.N., Rahman M.M. (2023). Impact of institutional quality on ecological footprint: new insights from G20 countries. J. Clean. Prod..

[32] Fatima N., Zheng Y., Guohua N. (2022). Globalization, institutional quality, economic growth and CO2 emission in OECD countries: an analysis with GMM and quantile regression. Front. Environ. Sci..

[33] Hunjra A.I., Tayachi T., Chani M.I., Verhoeven P., Mehmood A. (2020). The moderating effect of institutional quality on the financial development and environmental quality nexus. Sustain. Times.

[34] Abaidoo R., Agyapong E.K. (2022). Environmental sustainability risk, institutional effectiveness and urbanization. Energy Environ..

[35] Uzar U. (2021). The relationship between institutional quality and ecological footprint: is there a connection?. Nat. Resour. Forum.

[36] Shahbaz M., Dogan M., Akkus H.T., Gursoy S. (2023). The effect of financial development and economic growth on ecological footprint: evidence from top 10 emitter countries. Environ. Sci. Pollut. Res..

[37] Lee H.S., Chia C.J., Liew P.X., Lee S.Y., Har W.M. (2023).

[38] Majeed M.T., Tauqir A., Mazhar M., Samreen I. (2021). Asymmetric effects of energy consumption and economic growth on ecological footprint: new evidence from Pakistan. Environ. Sci. Pollut. Res..

[39] Iorember P.T., Gbaka S., Işık A., Nwani C., Abbas J. (2023). New insight into decoupling carbon emissions from economic growth: do financialization, human capital, and energy security risk matter?. Rev. Dev. Econ..

[40] Çakmak E.E., Acar S. (2022). The nexus between economic growth, renewable energy and ecological footprint: an empirical evidence from most oil-producing countries. J. Clean. Prod..

[41] Vu T.L., Paramaiah C., Tufail B., Nawaz M.A., Xuyen N.T.M., Huy P.Q. (2023). Effect of financial inclusion, eco-innovation, globalization, and sustainable economic growth on ecological footprint. Eng. Econ..

[42] Acar S., Altıntaş N., Haziyev V. (2023). The effect of financial development and economic growth on ecological footprint in Azerbaijan: an ARDL bound test approach with structural breaks. Environ. Ecol. Stat..

[43] Udemba E.N. (2020). A sustainable study of economic growth and development amidst ecological footprint: new insight from Nigerian Perspective. Sci. Total Environ..

[44] Weber H., Sciubba J.D. (2019). The effect of population growth on the environment: evidence from European regions. Eur. J. Popul..

[45] Rahman M.M., Hussian M.U.l., Azimi M.N. (2024). An environmental perspective of energy consumption, overpopulation, and human capital barriers in South Asia. Sci. Rep..

[46] Dimnwobi S.K., Ekesiobi C., Madichie C.V., Asongu S.A. (2021). Population dynamics and environmental quality in Africa. Sci. Total Environ..

[47] Kongbuamai N., Zafar M.W., Zaidi S.A.H., Liu Y. (2020). Determinants of the ecological footprint in Thailand: the influences of tourism, trade openness, and population density. Environ. Sci. Pollut. Res..

[48] Hussain M., Usman M., Khan J.A., Tarar Z.H., Sarwar M.A. (2022). Reinvestigation of environmental Kuznets curve with ecological footprints: empirical analysis of economic growth and population density. J. Publ. Aff..

[49] Anser M.K., Yousaf Z., Nassani A.A., Abro M.M.Q., Zaman K., Kabbani A. (2020). Evaluating ecological footprints through inbound tourism, population density, and global trade. Pol. J. Environ. Stud..

[50] World Bank (2020). List of Economies.

[51] Koengkan M., Fuinhas J.A., Santiago R. (2020). Asymmetric impacts of globalisation on CO2 emissions of countries in Latin America and the Caribbean. Environ. Syst. Decis..

[52] Jebabli I., Lahiani A., Mefteh-Wali S. (2023). Quantile connectedness between CO2 emissions and economic growth in G7 countries. Resour. Pol..

[53] Wang Q., Xiao K., Lu Z. (2020). Does economic policy uncertainty affect CO2 emissions? Empirical evidence from the United States. Sustain. Times.

[54] Koengkan M., Fuinhas J.A. (2020). Exploring the effect of the renewable energy transition on CO2 emissions of Latin American & Caribbean countries. Int. J. Sustain. Energy.

[55] Kazemzadeh E., Lotfalipour M.R., Shirazi M., Sargolzaie A. (2023). Heterogeneous effects of energy consumption structure on ecological footprint. Environ. Sci. Pollut. Res..

[56] Rehman A., Radulescu M., Ma H., Dagar V., Hussain I., Khan M.K. (2021). The impact of globalization, energy use, and trade on ecological footprint in Pakistan: does environmental sustainability exist?. Energies.

[57] Apaydin Ş., Ursavaş U., Koç Ü. (2021). The impact of globalization on the ecological footprint: do convergence clubs matter?. Environ. Sci. Pollut. Res..

[58] Omoke P.C., Nwani C., Effiong E.L., Evbuomwan O.O., Emenekwe C.C. (2020). The impact of financial development on carbon, non-carbon, and total ecological footprint in Nigeria: new evidence from asymmetric dynamic analysis. Environ. Sci. Pollut. Res..

[59] Alola A.A., Bekun F.V., Sarkodie S.A. (2019). Dynamic impact of trade policy, economic growth, fertility rate, renewable and non-renewable energy consumption on ecological footprint in Europe. Sci. Total Environ..

[60] Georgiou C. (2023). Federal fiscal capacity and the challenge of the green transition in the EU. J. Eur. Integrat..

[61] Ouedraogo Idrissa, Ngoa Tabi Henri, Atangana Ondoa Henri, Alex N.J. (2022). Institutional quality and human capital development in Africa. Econ. Syst..

[62] Iskandar I. (2017). Effect of human development index fund on economic growth through A special autonomy. J. Ekon. Pembang. Kaji. Masal. Ekon. dan Pembang..

[63] Yolanda Y. (2017). Analysis of factors affecting inflation and its impact on human development index and poverty in Indonesia. Eur. Res. Stud. J..

[64] Khan N.H., Ju Y., Hassan S.T. (2018). Modeling the impact of economic growth and terrorism on the human development index: collecting evidence from Pakistan. Environ. Sci. Pollut. Res..

[65] Iqbal K., Hassan S.T., Peng H., Khurshaid (2019). Analyzing the role of information and telecommunication technology in human development: panel data analysis. Environ. Sci. Pollut. Res..

[66] Sarma M. (2012). Index of financial inclusion – a measure of financial sector inclusiveness. Berlin Work. Pap. Money, Financ. Trade Dev..

[67] Azimi M.N. (Nov. 2022). New insights into the impact of financial inclusion on economic growth: a global perspective. PLoS One.

[68] Omar M.A., Inaba K. (2020). Does financial inclusion reduce poverty and income inequality in developing countries? A panel data analysis. J. Econ. Struct..

[69] Park C.-Y., Mercado R.V. (2016). Financial Inclusion in Asia.

[70] Hameed M.A., Rahman M.M., Khanam R. (2023). The health consequences of civil wars: evidence from Afghanistan. BMC Publ. Health.

[71] Chen Y., Lee C.C., Chen M. (2022). Ecological footprint, human capital, and urbanization. Energy Environ..

[72] Ahmed Z., Zafar M.W., Ali S., Danish (2020). Linking urbanization, human capital, and the ecological footprint in G7 countries: an empirical analysis. Sustain. Cities Soc..

[73] Jahanger A., Usman M., Murshed M., Mahmood H., Balsalobre-Lorente D. (2022). The linkages between natural resources, human capital, globalization, economic growth, financial development, and ecological footprint: the moderating role of technological innovations. Resour. Pol..

[74] Ahmed Z., Zhang B., Cary M. (2021). Linking economic globalization, economic growth, financial development, and ecological footprint: evidence from symmetric and asymmetric ARDL. Ecol. Indicat..

[75] Baloch M.A., Zhang J., Iqbal K., Iqbal Z. (2019). The effect of financial development on ecological footprint in BRI countries: evidence from panel data estimation. Environ. Sci. Pollut. Res..

[76] Wang Q., Yang T., Li R., Wang X. (2023). Reexamining the impact of foreign direct investment on carbon emissions: does per capita GDP matter?. Humanit. Soc. Sci. Commun..

[77] Zubair A.O., Abdul Samad A.R., Dankumo A.M. (2020). Does gross domestic income, trade integration, FDI inflows, GDP, and capital reduces CO2 emissions? An empirical evidence from Nigeria. Curr. Res. Environ. Sustain..

[78] Saqib N., Ozturk I., Usman M., Sharif A., Razzaq A. (2023). Pollution Haven or Halo? How European countries leverage FDI, energy, and human capital to alleviate their ecological footprint. Gondwana Res..

[79] Mahadevan R., Sun Y. (2020). Effects of foreign direct investment on carbon emissions: evidence from China and its Belt and Road countries. J. Environ. Manag..

[80] GFN (2023). https://api.footprintnetwork.org/v1/data/all/2019/EFCpc.

[81] World Bank (2023). https://databank.worldbank.org/source/world-development-indicators.

[82] UNO-WIDER (2023). https://www.wider.unu.edu/data.

[83] UNDP (2023). https://hdr.undp.org/data-center/human-development-index#/indicies/HDI.

[84] Kaufmann D., Kraay A. (2018).

[85] Hussain M.M., Pal S., Villanthenkodath M.A. (2023). Towards sustainable development: the impact of transport infrastructure expenditure on the ecological footprint in India. Innov. Green Dev..

[86] Gill A.R., Riaz R., Ali M. (2023). The asymmetric impact of financial development on ecological footprint in Pakistan. Environ. Sci. Pollut. Res..

[87] Ashraf A., Nguyen C.P., Doytch N. (2022). The impact of financial development on ecological footprints of nations. J. Environ. Manag..

[88] Levine R., Zervos S. (1998). Stock markets, banks, and economic growth. Am. Econ. Rev..

[89] King R.G., Levine R. (1993). Finance and growth: schumpeter might be right. Q. J. Econ..

[90] Hansen B.E. (2000). Sample splitting and threshold estimation. Econometrica.

[91] El Khoury A.C., Savvides A. (2006). Openness in services trade and economic growth. Econ. Lett..

[92] Law S.H., Azman-Saini W.N.W., Ibrahim M.H. (2013).

[93] Falvey R., Foster N., Greenaway D. (2007). Relative backwardness, absorptive capacity and knowledge spillovers. Econ. Lett..

[94] Azman-Saini W.N.W., Law S.H., Ahmad A.H. (2010). FDI and economic growth: new evidence on the role of financial markets. Econ. Lett..

[95] Bai J. (1997). Estimating multiple breaks one at a time. Econom. Theor..

[96] Bai J., Perron P. (1998). Estimating and testing linear models with multiple structural changes. Econometrica.

[97] Caner M., Hansen B.E. (2004). Instrumental variable estimation of a threshold model. Econom. Theor..

[98] Blundell R., Bond S. (1998). Initial conditions and moment restrictions in dynamic panel data models. J. Econom..

[99] Wu J.Y., Hsu C.C. (2012). Foreign direct investment and income inequality: does the relationship vary with absorptive capacity?. Econ. Modell..

[100] Azman-Saini W.N.W., Law S.H., Ahmad A.H. (2010). FDI and economic growth: new evidence on the role of financial markets. Econ. Lett..

[101] Cohen J., Cohen P., West S.G., Aiken L.S. (2003). Outliers and multicollinearity diagnosing and solving regression problems II. Appl. Mult. Regression/Correlation Anal. Behav. Sci..

[102] Elith J. (2006). Novel methods improve prediction of species' distributions from occurrence data. Ecography.

[103] O'Brien R.M. (2007). A caution regarding rules of thumb for variance inflation factors. Qual. Quantity.

[104] Pesaran M.H. (2004).

[105] Pesaran M.H. (2007). A simple panel unit root test in the presence of cross-section dependence. J. Appl. Econom..

[106] Shahbaz M., Sarwar S., Chen W., Malik M.N. (2017). Dynamics of electricity consumption, oil price and economic growth: global perspective. Energy Pol..

[107] Azimi Mohammad Naim, Rahman Mohammad Mafizur, Nghiem S. (2023). Linking governance with environmental quality: a global perspective. Sci. Rep..

[108] Hansen B.E. (1999). Threshold effects in non-dynamic panels: estimation, testing, and inference. J. Econom..

[109] Gonzalo J., Pitarakis J.Y. (2002). Estimation and model selection based inference in single and multiple threshold models. J. Econom..

[110] Driscoll J.C., Kraay A.C. (1998). Consistent covariance matrix estimation with spatially dependent panel data. Rev. Econ. Stat..

[111] Li C., Lin T., Chen Y., Yan Y., Xu Z. (2022). Nonlinear impacts of renewable energy consumption on economic growth and environmental pollution across China. J. Clean. Prod..

[112] Ullah A., Ahmed M., Raza S.A., Ali S. (2021). A threshold approach to sustainable development: nonlinear relationship between renewable energy consumption, natural resource rent, and ecological footprint. J. Environ. Manag..

[113] Shabir M., Pazienza P., De Lucia C. (2023). Energy innovation and ecological footprint: evidence from OECD countries during 1990–2018. Technol. Forecast. Soc. Change.

[114] Lee C.C., Zhang J., Hou S. (2023). The impact of regional renewable energy development on environmental sustainability in China. Resour. Pol..

[115] Arif M., Gill A.R., Ali M. (2023). Analyzing the non-linear association between urbanization and ecological footprint: an empirical analysis. Environ. Sci. Pollut. Res..

[116] Okelele D.O., Lokina R. (2022). Effect of trade openness on ecological footprint in sub-Saharan Africa. African J. Econ. Rev..

[117] Cutcu I., Beyaz A., Gerlikhan S.G., Kilic Y. (2023). Is ecological footprint related to foreign trade? Evidence from the top ten fastest developing countries in the global economy. J. Clean. Prod..

[118] Adebayo T.S., Sevinç H., Sevinç D.E., Ojekemi O.S., Kirikkaleli D. (2023). A wavelet-based model of trade openness with ecological footprint in the MINT economies. Energy Environ..

[119] Wang Q., Yang T., Li R. (2023). Economic complexity and ecological footprint: the role of energy structure, industrial structure, and labor force. J. Clean. Prod..

[120] Yang B., Usman M., Jahanger A. (2021). Do industrialization, economic growth and globalization processes influence the ecological footprint and healthcare expenditures? Fresh insights based on the STIRPAT model for countries with the highest healthcare expenditures. Sustain. Prod. Consum..

[121] Khan S.A.R., Yu Z., Belhadi A., Mardani A. (2020). Investigating the effects of renewable energy on international trade and environmental quality. J. Environ. Manag..

[122] Lakshmana S.B. (2023). Padhan, “Nexus between foreign direct investment and ecological footprint in BRICS and Next-11: the moderating role of green innovation,”. Manag. Environ. Qual..

[123] Khan H., Weili L., Khan I. (2022). Institutional quality, financial development and the influence of environmental factors on carbon emissions: evidence from a global perspective. Environ. Sci. Pollut. Res..

[124] Salahuddin M., Alam K., Ozturk I., Sohag K. (2018). The effects of electricity consumption, economic growth, financial development and foreign direct investment on CO2 emissions in Kuwait. Renew. Sustain. Energy Rev..

[125] Shahbaz M., Nasir M.A., Roubaud D. (2018). Environmental degradation in France: the effects of FDI, financial development, and energy innovations. Energy Econ..

[126] Stylianou T., Nasir R., Waqas M. (2023). Inclusive human development and governance nexus: causality analysis of selected Asian countries. Economies.

[127] Pata U.K., Aydin M., Haouas I. (2021). Are natural resources abundance and human development a solution for environmental pressure? Evidence from top ten countries with the largest ecological footprint. Resour. Pol..

[128] Sultana N., Rahman M.M., Khanam R. (2022). Environmental kuznets curve and causal links between environmental degradation and selected socioeconomic indicators in Bangladesh. Environ. Dev. Sustain..

[129] Ünal H., Aktuğ M. (2022). The impact of human capital and bio-capacity on the environmental quality: evidence from G20 countries. Environ. Sci. Pollut. Res..

[130] Pata U.K., Hizarci A.E. (2022). Investigating the environmental Kuznets curve in the five most complex countries: insights from a modified ecological footprint model. Energy Environ..

[131] Ohlan R. (2015). The impact of population density, energy consumption, economic growth and trade openness on CO2 emissions in India. Nat. Hazards.

[132] Egbetokun S., Osabuohien E., Akinbobola T., Onanuga O.T., Gershon O., Okafor V. (2020). Environmental pollution, economic growth and institutional quality: exploring the nexus in Nigeria. Manag. Environ. Qual. Int. J..

[133] Apergis N., Ozturk I. (2015). Testing environmental Kuznets curve hypothesis in Asian countries. Ecol. Indicat..

[134] Sapkota P., Bastola U. (2017). Foreign direct investment, income, and environmental pollution in developing countries: panel data analysis of Latin America. Energy Econ..

[135] Naimoglu M. (2023). The effect of energy prices, energy losses, and renewable energy use on CO2 emissions in energy-importing developing economies in the presence of an environmental Kuznets curve. Environ. Sci. Pollut. Res..

[136] Salahuddin M., Habib M.A., Al-Mulali U., Ozturk I., Marshall M., Ali M.I. (2020). Renewable energy and environmental quality: a second-generation panel evidence from the Sub Saharan Africa (SSA) countries. Environ. Res..

[137] Sinha A., Shahbaz M. (2018). Estimation of environmental Kuznets curve for CO2 emission: role of renewable energy generation in India. Renew. Energy.

[138] Sugiawan Y., Managi S. (2016). The environmental Kuznets curve in Indonesia: exploring the potential of renewable energy. Energy Pol..

[139] Danish, Ulucak R. (2020). How do environmental technologies affect green growth? Evidence from BRICS economies. Sci. Total Environ..

[140] Alola A.A., Yalçiner K., Alola U.V., Saint Akadiri S. (2019). The role of renewable energy, immigration and real income in environmental sustainability target. Evidence from Europe largest states. Sci. Total Environ..

[141] Qiao H., Zheng F., Jiang H., Dong K. (2019). The greenhouse effect of the agriculture-economic growth-renewable energy nexus: evidence from G20 countries. Sci. Total Environ..

[142] Adams S., Nsiah C. (2019). Reducing carbon dioxide emissions; Does renewable energy matter?. Sci. Total Environ..

[143] Diallo S. (2023). Effect of renewable energy consumption on environmental quality in sub-Saharan African countries: evidence from defactored instrumental variables method. Manag. Environ. Qual..

[144] Hausman J.A. (1978). Specification tests in econometrics. Econometrica.

[145] Ahmad M., Satrovic E. (2023). How do fiscal policy, technological innovation, and economic openness expedite environmental sustainability?. Gondwana Res..

[146] Lan J., Munro A. (2013). Environmental compliance and human capital: evidence from Chinese industrial firms. Resour. Energy Econ..

[147] Abdouli M., Omri A. (2021). Exploring the nexus among FDI inflows, environmental quality, human capital, and economic growth in the editerranean region. J. Knowl. Econ..

[148] Çakar N.D., Gedikli A., Erdoğan S., Yıldırım D.Ç. (2021). Exploring the nexus between human capital and environmental degradation: the case of EU countries. J. Environ. Manag..

[149] Jain M., Nagpal A. (2019). Relationship between environmental sustainability and human development index: a case of selected South Asian nations. Vision.

[150] Bongers A. (2022). Energy mix, technological change, and the environment. Environ. Econ. Pol. Stud..

[151] Sutiah and Supriyono (2021). Environment, mix energies, asean economies and education. Int. J. Energy Econ. Pol..

[152] El Anshasy A.A., Katsaiti M.S. (2014). Energy intensity and the energy mix: what works for the environment?. J. Environ. Manag..

[153] Christoforidis T., Katrakilidis C. (2021). The dynamic role of institutional quality, renewable and non-renewable energy on the ecological footprint of OECD countries: do institutions and renewables function as leverage points for environmental sustainability?. Environ. Sci. Pollut. Res..

[154] Hussain M., Dogan E. (2021). The role of institutional quality and environment-related technologies in environmental degradation for BRICS. J. Clean. Prod..

[155] Xu J., Moslehpour M., Tran T.K., Dinh K.C., Ngo T.Q., Huy P.Q. (2023). The role of institutional quality, renewable energy development and trade openness in green finance: empirical evidence from South Asian countries. Renew. Energy.

[156] Nadee M., Malik M.I., Adil S., Junaid N. (2023). Exploring the determinants of ecological efficiency in selected emerging economies using pooled mean group estimator. Reg. Stat.

