# Supplementary material for: Renewable energy and ecological footprint nexus: Evidence from dynamic panel threshold technique
Source: Heliyon. 2024 Jun 22;10(13):e33442. doi: 10.1016/j.heliyon.2024.e33442 (PMC11255664; doi:10.1016/j.heliyon.2024.e33442)
Supplement: Multimedia component 1 [file mmc1.docx]

**Supplementary information**

*Appendix A*

**IQI construction procedure**

We construct the aggregate institutional quality index (IQI) using the proposed method by Sarma (2012) from six measures of the WGI. These measures include voice and accountability, political stability, regulatory quality, control of corruption, government effectiveness, and the rule of law. The construction of IQI is carried out in three steps as follows:

**Step one**

First, we collect the required data for the above-mentioned indicators WGI’s sources. These indicators are expressed in percentile ranks (ranging from 1 to 100). Without standardizing the indicators, as they are all reflected by similar units of measurement, we assign the indicators with an appropriate index value using equation 1a as follows:

$\Omega_{i}=\sum_{i=1}^{n} \left(\frac{Ac_{i}-Lo_{i}}{Hg_{i}-Lo_{i}}\right) w_{i}$ (1a)

where, in equation (1), $\Omega_{i}=$ estimated index values, ${Ac}_{i}=$ actual value, ${LO}_{i}=$ lower values (set to be 0), ${Hg}_{i}=$ higher values (set to be 80^th^ percentile) to capture any variability of the indicators across time and units, and $w_{i}=$ allocated weights of the indicators that were estimated using the coefficient of variations over standard error of the WGI indicators.

**Step two**

Next, we estimate the normalized- and inverse-normalized Euclidian values for the indicators to determine the distance points from the worse point to an ideal point using equations (2a and 3a) as follows:

$X_{i}^{norm}=\sqrt{\left(\sum_{i=1}^{n} \Omega_{i}^{2}\right)}/\sqrt{\left(\sum_{i=1}^{n} w_{i}^{2}\right)}$ (2a)

$X_{i}^{inorm}=1-\left(\sqrt{\left(\sum_{i=1}^{n} \left(w_{i}-\Omega_{it}\right)^{2}\right)}/\sqrt{\left(\sum_{i=1}^{n} w_{i}^{2}\right)}\right)$ (3a)

where $X_{i}^{norm}=$ normalized Euclidian and $X_{i}^{inorm}=$ inverse-normalized Euclidian values.

**Step three**

Using equations (2a) and (3a), we can construct the IQI as follows:

$IQI_{i}=\frac{1}{2}\left(X_{i}^{norm}+X_{i}^{inorm}\right)$ (4a)

where the constructed IQI is expressed in numbers ranging between 0 and 1 (high).
